# Novel Roles for Peroxynitrite in Angiotensin II and CaMKII Signaling

**DOI:** 10.1038/srep23416

**Published:** 2016-04-15

**Authors:** Chaoming Zhou, Swarna S. Ramaswamy, Derrick E. Johnson, Dario A. Vitturi, Franciso J. Schopfer, Bruce A. Freeman, Andy Hudmon, Edwin S. Levitan

**Affiliations:** 1 Department of Pharmacology and Chemical Biology, University of Pittsburgh School of Medicine, 200 Lothrop Street, Pittsburgh, PA 15261 USA; 2 Department of Biochemistry and Molecular Biology and Stark Neuroscience Research Institute, Indiana University School of Medicine, 320 West 15th Street, Indianapolis, IN 46202 USA

## Abstract

Ca^2+^/calmodulin-dependent protein kinase II (CaMKII) oxidation controls excitability and viability. While hydrogen peroxide (H_2_O_2_) affects Ca^2+^-activated CaMKII *in vitro*, Angiotensin II (Ang II)-induced CaMKIIδ signaling in cardiomyocytes is Ca^2+^ independent and requires NADPH oxidase-derived superoxide, but not its dismutation product H_2_O_2_. To better define the biological regulation of CaMKII activation and signaling by Ang II, we evaluated the potential for peroxynitrite (ONOO^−^) to mediate CaMKII activation and downstream Kv4.3 channel mRNA destabilization by Ang II. *In vitro* experiments show that ONOO^−^ oxidizes and modestly activates pure CaMKII in the absence of Ca^2+^/CaM. Remarkably, this apokinase stimulation persists after mutating known oxidation targets (M281, M282, C290), suggesting a novel mechanism for increasing baseline Ca^2+^-independent CaMKII activity. The role of ONOO^−^ in cardiac and neuronal responses to Ang II was then tested by scavenging ONOO^−^ and preventing its formation by inhibiting nitric oxide synthase. Both treatments blocked Ang II effects on Kv4.3, tyrosine nitration and CaMKIIδ oxidation and activation. Together, these data show that ONOO^−^ participates in Ang II-CaMKII signaling. The requirement for ONOO^−^ in transducing Ang II signaling identifies ONOO^−^, which has been viewed as a reactive damaging byproduct of superoxide and nitric oxide, as a mediator of GPCR-CaMKII signaling.

Angiotensin II (Ang II) induces NADPH oxidase-dependent generation of partially reduced oxygen species (superoxide, O_2_^.−^; hydrogen peroxide, H_2_O_2_) to regulate CaMKIIδ activity and promote apoptosis, hypertrophy and downregulation of Kv4.3 K^+^ channels in cardiomyocytes^1^,^2^,^3^,^4^,^5^,^6^. The latter effect is also produced in neurons, possibly to contribute to hypothalamic function^7^. *In vitro* studies originally used H_2_O_2_, the dismutation product of O_2_^.−^, to show autoinhibitory domain methionine oxidation (i.e., at M281/282), which sustains CaMKII activity after Ca^2+^ is removed and CaM is dissociated (i.e., the enzyme becomes autonomous after Ca^2+^ priming)^1^. However, Ang II-induced cardiomyocyte hypertrophy and Kv4.3 downregulation are inhibited by superoxide dismutase (SOD) but not by catalase, suggesting that O_2_^.−^, rather than H_2_O_2_, is required for Ang II signaling^3^,^4^,^5^. Furthermore, Ang II activation of CaMKII in cardiomyocytes is independent of Ca^2+^ and persists in the presence of a CaM inhibitor^6^, which again contrasts with the *in vitro* requirement for Ca^2+^/CaM priming for H_2_O_2_-mediated generation of autonomous activity^1^,^2^. Thus, these findings exclude H_2_O_2_ as the mediator of Ang II-CaMKII signaling and demonstrate the involvement of O_2_^.−^. Similarly, nitric oxide (NO) cannot on its own account for Ang II effects as SOD would not be inhibitory. However, O_2_^.−^ is not a robust oxidizing species and does not react with methionine, which is oxidized in CaMKII in response to Ang II^1^. Therefore, it has been puzzling how O_2_^.−^ mediates Ang II signaling.

One possibility is that O_2_^.−^ is a precursor for a more reactive species. A candidate reactive species is peroxynitrite (ONOO^−^), which is generated by the diffusion-limited reaction of O_2_^.−^ and NO^8^,^9^. Previously, synthetic ONOO^−^ was shown to react with pure Ca^2+^/CaM-activated CaMKII *in vitro* to generate Ca^2+^-primed autonomous activity^10^,^11^. For CaMKIIδ, this autonomy requires M281/282^11^. These studies had limitations, however, in that they did not (a) measure CaMKII methionine oxidation as only activity was assayed; (b) distinguish whether ONOO^−^ or contaminants from its synthesis or spontaneous decomposition account for CaMKII responses; (c) determine if CaMKII regulation strictly depends on Ca^2+^/CaM activation; and (d) address whether ONOO^−^ is required for Ang II stimulation of CaMKII in living cells. Therefore, we tested the hypothesis that ONOO^−^ participates in Ang II-induced CaMKII signaling by conducting experiments with purified CaMKII and cardiac and neuronal cells.

## Results

### ONOO^−^-induced oxidation and activation of purified apoCaMKIIδ

**Figure 1:**
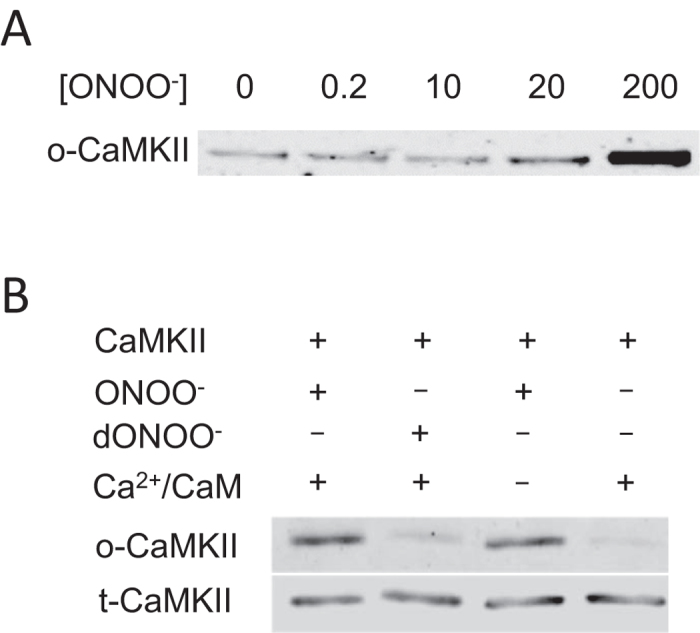
ONOO^−^ induces methionine oxidation of purified recombinant CaMKIIδ *in vitro*. (**A**) M281/282 oxidized CaMKII (o-CaMKII) immunoblot showing concentration dependent CaMKIIδ methionine oxidation by ONOO^−^. Numbers are micromolar concentrations. Ca^2+^ and CaM were present. B. CaMKIIδ M281/282 oxidation by 200 μM ONOO^−^ (but not 200 μM dONOO^−^) in the presence and absence of Ca^2+^ and CaM. t-CaMKII, total CaMKII.

Previous *in vitro* experiments with ONOO^−^ and purified CaMKIIδ reported increased autonomous activity following Ca^2+^ priming^10^,^11^, an effect we have reproduced with human CaMKIIδ ONOO^−^ (27.64% ± 1.53%). To extend these studies, the effect of ONOO^−^ on CaMKIIδ methionine oxidation was determined and compared with a control consisting of neutral pH decomposition products and potential residual species used in the synthesis of ONOO^−^ (nitrite (NO_2_^−^), nitrate (NO_3_^−^), H_2_O_2_). First, recombinant human CaMKIIδ was activated by Ca^2+^ and CaM in the absence of Mg^2+^ and ATP and then treated with ONOO^−^. CaMKIIδ M281/282 oxidation state was assayed by immunoblot using a site-specific redox antibody. A low signal for M281/282 oxidation was observed for no ONOO^−^ treatment and treatment with low concentrations of ONOO^−^ (0.2 and 2 μM), while 20 and 200 μM ONOO^−^ enhanced methionine oxidation of CaMKIIδ (Fig. 1A), demonstrating concentration dependent redox modification of CaMKII expose to ONOO^−^. The relatively high concentrations of ONOO^−^ required to induce CaMKIIδ oxidation *in vitro* are possibly due to the short half-life of ONOO^−^ at neutral pH (~1 second)^8^,^9^ and competing reactions of other reaction mixture constituents including residual β-ME present in the purified enzyme preparation. To distinguish whether the increase in methionine oxidation was mediated by ONOO^−^, as opposed to decay products or contaminants from synthesis, ONOO^−^ was added to neutral buffer for 2–5 minutes before exposing these products to CaMKIIδ. In contrast to ONOO^−^, decayed ONOO^−^ (dONOO^−^) resulted in no CaMKIIδ methionine 281/282 oxidation (Fig. 1B). Thus, M281/282 oxidation of CaMKII supports the conclusion that ONOO^−^ is a candidate mediator for the modulation of CaMKIIδ activity in cells.

Based on cellular results showing Ca^2+^-independent CaMKII activation^6^, we considered whether ONOO^−^ could induce CaMKII oxidation in the absence Ca^2+^ and CaM (i.e., with the apoenzyme). Remarkably, immunoblotting showed that CaMKIIδ M281/282 oxidation induced by ONOO^−^ was similar in the presence and absence of Ca^2+^ and CaM (Fig. 1B). While previous studies have largely focused on activity-dependent changes requiring priming, these experiments clearly show that ONOO^−^-induced oxidation does not require Ca^2+^-CaM binding to permit CaMKII oxidation in the autoregulatory domain (ARD).

**Figure 2:**
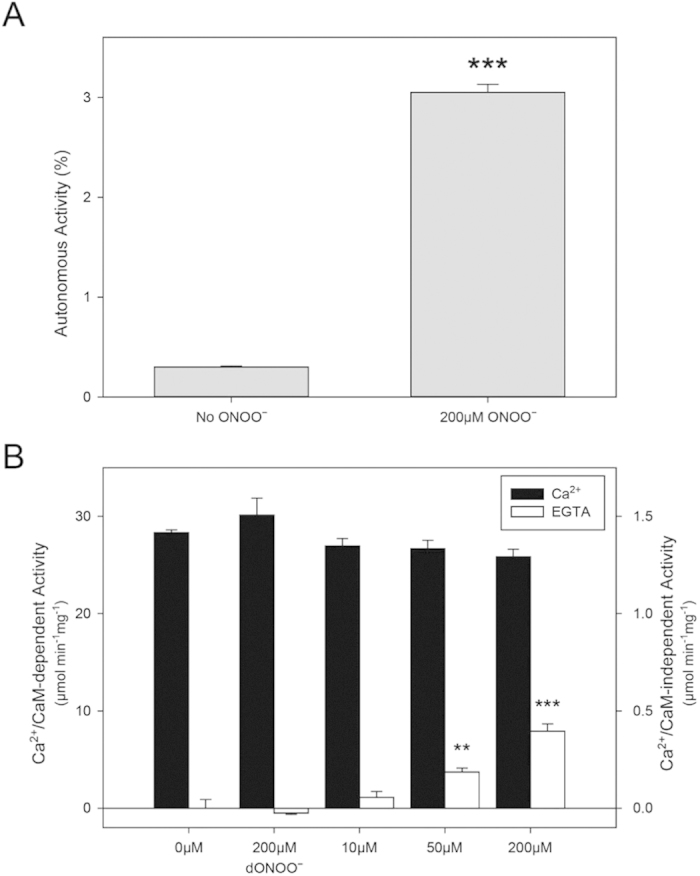
ONOO^−^ induced activation in CAMKIIδ. (**A**) Percentage of autonomous activity of CaMKIIδ in the presence and absence of 200 μM ONOO^−^. The percentage was calculated from activity of apoenzyme in the presence of EGTA in comparison to maximal activity in the presence of Ca^2+^/CaM. One-way ANOVA using Dunnett’s test in comparison to control (No ONOO^−^); ***p < 0.001 (n = 5). (**B**) Kinase activity of CaMKIIδ in the presence of Ca^2+^/CaM (Ca^2+^/CaM-dependent activity) and EGTA (Ca^2+^/CaM independent activity) with increasing concentrations of ONOO^−^ and dONOO^−^. Activity is represented as specific activity, in μmol min^−1^ mg^−1^. Data represent mean values ± s.e.m from 3 experiments. Note the differences in scale for the two y-axes. One-way ANOVA using Dunnett’s post-test compared with control (No ONOO^−^); **p < 0.01; ***p < 0.001.

This result led us to consider that CaMKII oxidation in the absence of Ca^2+^/CaM may lead to activity independent of Ca^2+^/CaM priming. The effect of ONOO^−^ on CaMKIIδ apoenzyme was therefore determined by studying enzymatic activity under low Ca^2+^ (5 mM EGTA, no CaM) conditions. Despite preventing canonical activation by omitting CaM and chelating Ca^2+^, ONOO^−^ (but not dONOO^−^) induced a small, yet significant increase in autonomous activity in freshly isolated enzyme (Fig. 2A, n = 5, p < 0.001). We observed minimal loss of Ca^2+^/CaM-dependent activation of CaMKII with ONOO^−^ oxidation, while detecting a ONOO^−^ dose-dependent increase in apoenzyme activity (Fig. 2B). Specifically, the increase in apoCaMKIIδ activity at concentrations 200 μM and 50 μM of ONOO^−^ is significantly different compared to the reaction without ONOO^−^ (p < 0.001 and p < 0.01, respectively). Thus, these experimental data show that ONOO^−^ induces a small, yet significant increase in apoCaMKIIδ activation without a requirement for Ca^2+^ and CaM in either the oxidation step or to increase basal activity by prior disinhibition of the ARD.

The ONOO^−^-induced apoCaMKII activity *in vitro* is less than seen with priming in saturating Ca^2+^/CaM (i.e., ~9-fold difference). Nevertheless, this increase in apoCaMKII activity could be biologically relevant. First, because maximal activation is likely never attained in cells, normalization to maximal activity is not a representation of physiological activity. Second, a small degree of apoCaMKII activation could lead to autophosphorylation, which would amplify Ca^2+^-induced activation. Third, basal activation is sustained and so not limited by the incidence of action potentials. Therefore, enhanced modest activation could be functionally significant over a sustained period of time. Finally, increased basal activity plus the enhanced autonomous activity previously reported following Ca^2+^/CaM priming^10^,^11^ could interact to contribute to ONOO^−^-induced signaling by CaMKII. Therefore, we explored the molecular basis of ONOO^−^ action on apoCaMKII.

### Direct ONOO^−^ activation of CaMKII is not mediated by M281, M282 or C290

Because prior experiments demonstrated roles for M281/282 and C290 in facilitation of Ca^2+^-CaM-primed CaMKII activity by H_2_O_2_, ONOO^−^ and NO^1^,^10^,^11^,^12^, we examined whether these residues are required for the ONOO^−^ effect on wildtype human apoCaMKIIδ activity using double (M281V/M282V) and triple (M281V/M282V/C290V) mutants of these known oxidation sites in the ARD. To minimize complications with CaMKIIδ oxidation during storage and freezing, we expressed and purified the wildtype, double and triple mutants simultaneously and used fresh non-frozen enzyme to perform the comparisons.

**Figure 3:**
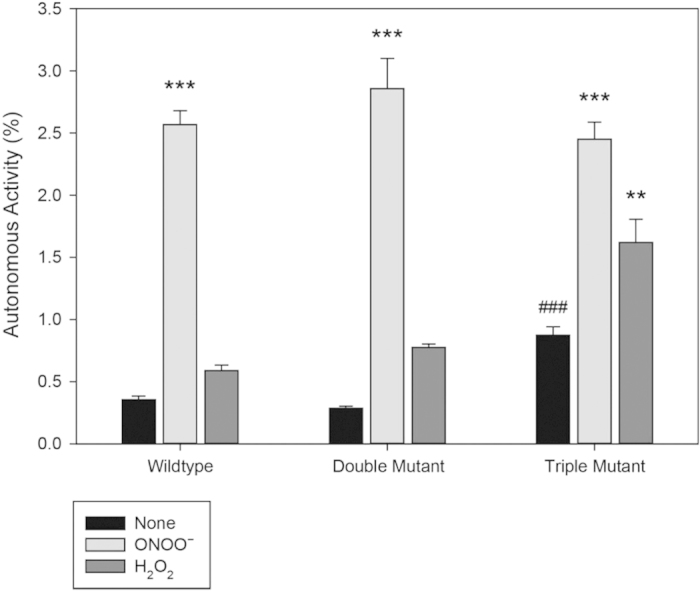
Direct ONOO^−^ activation of apoCaMKII is not mediated by M281/282 or C290. Ca^2+^/CaM-independent activity is plotted as a percentage of the Ca^2+^/CaM dependent activity for WT, double and triple oxidation mutants. The % autonomy was calculated for the wildtype, double (MM281,282VV) and triple (MM281,282VV; C290V) mutants in the following conditions: no oxidation (None); with 200 μM ONOO^−^ and with 3 mM H_2_O_2_. One-way ANOVA using Dunnett’s test compared to controls- **p < 0.01; ***p < 0.001- significance of difference upon oxidation compared to no oxidation; ^###^p < 0.001- significance of mutation compared to wildtype.

In the absence of oxidizers, wildtype and the double mutant displayed similar apoCaMKII activity. However, the triple mutant displayed enhanced apoCaMKII activity (Fig. 3, black bars), suggesting that the triple mutant “loosens” the ARD compared to wildtype and the double mutant of CaMKIIδ. More importantly, ONOO^−^ activation of CaMKII was similar for wildtype and the double and triple mutants (Fig. 3, light gray bars). Therefore, previously identified ARD oxidation sites that are important for regulating Ca^2+^/CaM-primed CaMKII are not required for ONOO^−^-induced activation of apoCaMKII.

This surprising result led us to examine the effect of H_2_O_2_ (Fig. 3, dark gray bars), which was originally used to demonstrate regulation of Ca^2+^/CaM-primed CaMKII by methionine oxidation^1^. When the effects of ONOO^−^ and H_2_O_2_ were compared in side-by-side studies, we observed that, as described previously, H_2_O_2_ did not induce apoCaMKII activity with the wildtype enzyme. In contrast, the triple mutant did appear susceptible to a statistically significant increase in H_2_O_2_ activation compared to wildtype. However, the autonomous activity generated by H_2_O_2_ did not reach the same level as seen by ONOO^−^ even though a 15-fold higher concentration was used (3 mM) and H_2_O_2_ is much more stable than ONOO^−^, which decomposes in seconds. Therefore, the novel Ca^2+^-independent increase in apoenzyme activity described here must involve a residue that has not been previously implicated in redox regulation of Ca^2+^/CaM-primed CaMKII. Apparently, this site is readily oxidized by ONOO^−^, but less reactive H_2_O_2_ is not effective unless the enzyme is partially activated by mutagenesis of the ARD.

### Ang II induces ONOO^−^ signaling in cardiac and neuronal cells

**Figure 4:**
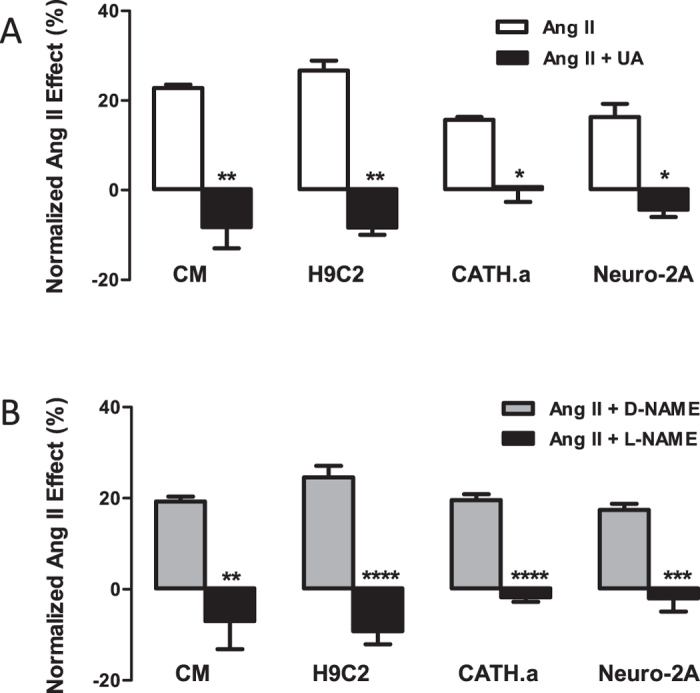
ONOO^−^ is required for Ang II-Kv4.3 signaling in cardiac and neural cells. (**A**) Kv4.3 luciferase reporter response to 100 nM Ang II in cardiomyocytes (CM), H9C2 cells, Catha.a cells and Neuro-2A cells in the presence or absence of 300 μM UA. Each bars represent mean values ± s.e.m. from 4–9 experiments. Ang II alone in each cell type, p < 0.001. (**B**) L-NAME, but not D-NAME, inhibits the Ang II-Kv4.3 signaling in cardiac and neural cells (cardiomyocytes (CM), H9C2 cells, CATH.a cells, and N2A cells). Data are mean values ± s.e.m. from 4–7 luciferase reporter experiments. *p < 0.05; **p < 0.01; ***p < 0.001; ****p < 0.0001.

Contrary to prior reports, the above *in vitro* studies using human CaMKIIδ are consistent with the Ca^2+^-independent activation described previously in cardiomyocytes^6^ and so support the hypothesis that ONOO^−^ may contribute to Ang II-induced activation of CaMKII in cells, at least in part through Ca^2+^-independent modifications. Thus, to examine the mechanisms underlying Ang-II redox signaling, we turned to the Ang II-induced CaMKII activation shown previously to downregulate the Kv4.3 K^+^ channel, which is produced by mRNA destabilization induced by AUF1 protein binding to the Kv4.3 3′ untranslated region (3′ UTR) in response to CaMKII phosphorylation^2^,^5^,^13^. Using the Kv4.3 3′ UTR luciferase reporter construct^5^, we evaluated upstream redox signaling regulating Kv4.3 mRNA stability. Although the turnover of luciferase attenuates the effect on native mRNA, internal normalization ensures that results are quantitative and reproducible and are not impacted by presence of native Kv4.3 mRNA. Therefore, the reporter was transfected into cultured neonatal rat ventricular cardiomyocytes (CM), cardiac H9C2 cells, neuronal CATH.a and Neuro-2a cells. In both cardiac and neuronal backgrounds, reporter activity was sensitive to 100 nM Ang II (Fig. 4A, open bars).

Because this suggests shared signaling, the four cell systems were also used to test whether the Ang II-Kv4.3 effect requires ONOO^−^. First, the impact of the ONOO^−^ scavenger FeTPPS (5,10,15,20-Tetrakis(4-sulfonatophenyl) porphyrinato Iron (III)) was determined. 50–100 μM FeTPPS blocked the Ang II-induced reporter responses, increased the baseline reporter signal and altered cell morphology, suggesting possible toxicity. To address this limitation, the ONOO^−^ scavenger UA was used^10^. UA did not affect baseline values or morphology, but blocked the Ang II responses in the four cell systems (Fig. 4A). The four cell systems were then used to test whether the Ang II-Kv4.3 effect requires NO, which reacts with O_2_^.−^ to yield ONOO^−^14. Specifically, the effect of the NO synthesis inhibitor L-NAME was compared to its inactive isomer D-NAME. In all four cases, L-NAME, but not D-NAME, abolished the Ang II-Kv4.3 response (Fig. 4B). The requirement for O_2_^.−^ for this signaling^7^ and the above effect of scavenging ONOO^−^ with UA show that NO is not sufficient for this Ang II effect. Instead, these results suggest a requirement for ONOO^−^ in cardiac and neuronal cell Ang II-Kv4.3 mRNA responses.

**Figure 5:**
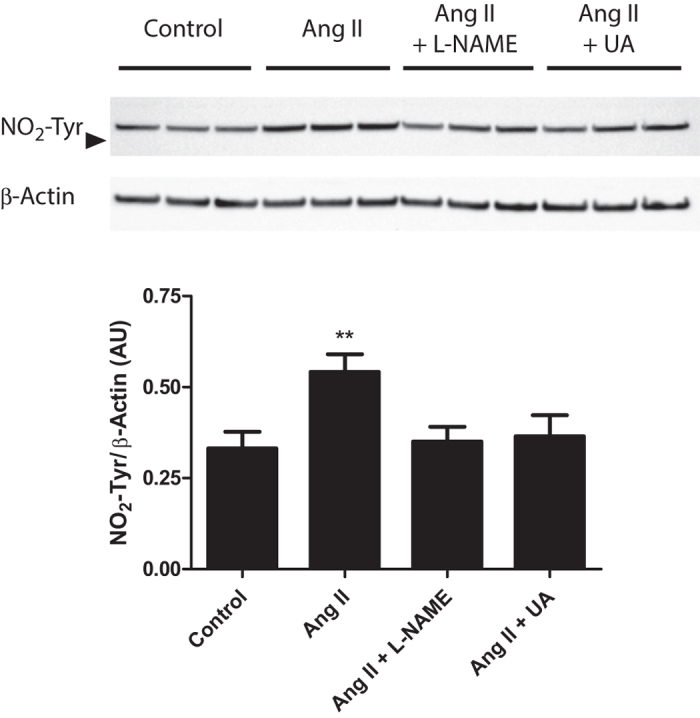
Ang II acts via ONOO^−^ to increase tyrosine nitration in H9C2 cells. Top, immunoblots showing nitrotyrosine signal (arrowhead shows 50 kDa) and β-actin, which was used for normalization. Bottom, quantification of the nitrotyrosine signal. Data are mean values ± s.e.m (n = 3). **p < 0.01.

ONOO^−^ becomes protonated under physiological conditions to peroxynitrous acid (ONOOH), which in turn undergoes homolytic scission to the oxidizing species hydroxyl radical (^.^OH) and the nitrating species nitrogen dioxide (^.^NO_2_). An index of ONOO^−^ generation and signaling is tyrosine nitration, which is induced by .NO_2_ and not the individual reactions of O_2_^.−^, H_2_O_2_ or NO. Therefore, to independently test whether Ang II signals via ONOO^−^, tyrosine nitration was assayed by immunoblot from H9C2 cell extracts by quantifying the formation of nitrotyrosine (NO_2_-Tyr) adducts. In these experiments, the strongest signal was from a 53 kD protein, which may be derived from desmin, a key target of tyrosine nitration in the heart^15^. Consistent with the generation and action of ONOO^−^, not only did Ang II induce tyrosine nitration in H9c2 cells but most importantly, this effect was abolished by either inhibition of NO synthesis with L-NAME or by the ONOO^−^ scavenger UA (Fig. 5). These results independently support the conclusion that Ang II promotes cellular ONOO^−^ generation.

**Figure 6:**
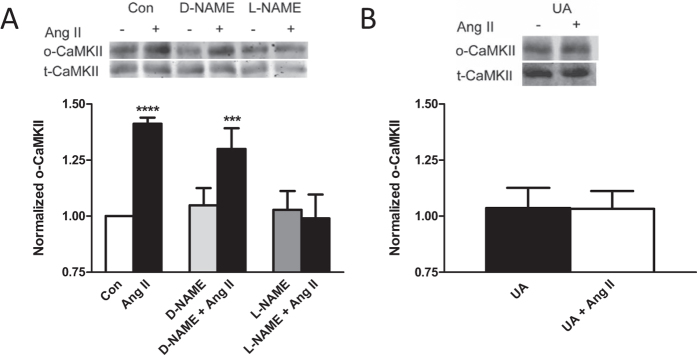
ONOO^−^ is required for Ang II-induced CaMKIIδ M281/282 oxidation in cardiomyocytes. (**A**) Ang II-induced M281/282 oxidation of CaMKIIδ is inhibited by L-NAME, but not by D-NAME. Data are mean values ± s.e.m. from at least 5 experiments. (**B**) Ang II-induced methionine oxidation of CaMKIIδ is inhibited by UA. Data are mean values ± s.e.m. from at least 5 experiments. ***p < 0.001; ****p < 0.0001.

It is known that Ang II induces oxidation of CaMKIIδ in cardiomyocytes, which has been measured by immunoblot with an antibody that detects M281/282 oxidation^1^,^2^. Therefore, cardiomyocytes were treated with L-NAME, D-NAME and UA and immunoprecipitated CaMKIIδ was probed for M281/282 oxidation. As can be seen in Fig. 6, disrupting ONOO^−^ signaling inhibited oxidation of CaMKII. Thus, these experiments further establish that Ang II induces ONOO^−^-mediated protein oxidation.

### Ang II-induced autophosphorylation of CaMKII requires ONOO^−^

**Figure 7:**
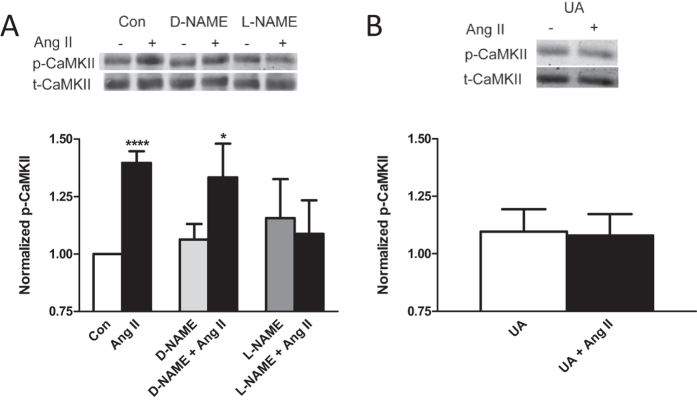
ONOO^−^ is required for Ang II-induced CaMKIIδ activation in cardiomyocytes. (**A**) Ang II-induced T287 autophosphorylation of CaMKIIδ (p-CaMKII) is inhibited by L-NAME, but not D-NAME. Data are mean values ± s.e.m. from at least 7 experiments. (**B**) Ang II-induced T287 CaMKIIδ autophosphorylation is inhibited by UA. Data are mean values ± s.e.m. from at least 6 experiments. *p < 0.05; ****p < 0.0001.

Ang II regulation of Kv4.3 mRNA stability requires a delayed prolonged phase of CaMKII signaling^2^. To test whether ONOO^−^ induces this effect, cardiomyocytes were treated for 150 min with Ang II, D-NAME, L-NAME, and/or UA, then cell extracts were generated and endogenous CaMKIIδ was immunoprecipitated. The effect on Ang II-induced CaMKIIδ enzymatic activation was then measured by assaying T287 autophosphorylation of CaMKIIδ by immunoblot, an established method that is sensitive enough to be applied to primary cultures containing thousands of cardiomyocytes. Consistent with the above results, CaMKIIδ autophosphorylation induced by Ang II was blocked by L-NAME, but not D-NAME (Fig. 7A). Furthermore, UA also inhibited Ang II-induced CaMKIIδ autophosphorylation (Fig. 7B), affirming that OONO^−^ is required for Ang II-induced CaMKII activation.

## Discussion

This study was motivated by a puzzle regarding the role of Ca^2+^ and redox signaling underlying Ang II action. Hypertrophy and Kv4.3 studies have established that O_2_^.−^, but not H_2_O_2_, is required for Ang II signaling^3^,^4^,^5^. Furthermore, CaMKII is required for Ang II-induced effects (e.g. on Kv4.3 expression and apoptosis), which are correlated with CaMKII methionine oxidation^1^,^2^. However, oxidative stimulation of CaMKII was thought to require priming by Ca^2+^, while Ang II can activate CaMKII independently of Ca^2+^ 6. Furthermore, O_2_^.−^ is a weak oxidizer of methionine. Therefore, we wondered how Ang II acts via O_2_^.−^ and independently of H_2_O_2_ and Ca^2+^ to stimulate CaMKII-induced Kv4.3 downregulation. Purified enzyme and cell-based CaMKIIδ activation studies reported herein resolve this quandary by showing that ONOO^−^ is necessary and sufficient for CaMKII activation by Ang II. Specifically, the involvement of O_2_^.−^ without dismutation to H_2_O_2_, is now understood in terms of the requirement of O_2_^.−^ as a precursor of ONOO^−^ generation. Therefore, the identification of ONOO^−^ as a signaling intermediate clarifies how Ang II activates CaMKII to control Kv4.3 expression.

Previous experiments have implicated methionines 281 and 282 and cysteine 290 in promoting autonomy of Ca^2+^-primed CaMKII^1^,^10^,^11^,^12^, but the current study shows that another mechanism operates independently of these residues to increase apoCaMKIIδ activity in response to ONOO^−^. This novel activation mechanism is a fraction of the activity compared to canonical Ca^2+^/CaM stimulation and smaller than ONOO^−^-induced autonomy following Ca^2+^/CaM priming. However, the relative impact of this change in basal activity induced by ONOO^−^ may be more significant than indicated by percentage of maximal activation measured *in vitro*. First, because bulk Ca^2+^ in cells never reaches levels required to saturate CaMKII under conditions of limiting free [CaM], autophosphorylation within the CaM-target domain of CaMKII can prevent CaM activation, and CaM itself is sensitive to oxidation, which renders it unable to activate CaMKII^16^,^17^,^18^,^19^, CaMKII signaling is likely submaximal under physiological conditions. The extent of ONOO^−^ activation may also be underestimated because the purified kinase may become oxidized during isolation and storage. In fact, our experience with the wildtype CaMKIIδ was that as the enzyme aged in the −80 °C freezer, the ability to induce ONOO^−^-induced apoenzyme activity diminished, which is why for the comparison to mutants we purified all of the material together to avoid aging artifacts. Finally, increased basal activation may synergize with conventional effects of Ca^2+^ and the Ca^2+^-dependent effects of ONOO^−^ and NO^10^,^11^,^12^,^20^. This may be particularly important in the context of prolonged Ang II signaling, which is important for controlling hypertrophy, channel expression and apoptosis. Thus, the apparently small change in apoCaMKII activity may be significant in living cells.

Ang II-induced activation and oxidation of CaMKII is biphasic with long lasting activation becoming evident after a delay of hours following transient activation^2^. Therefore, it is likely that ONOO^−^, which mediates Ang II action but is chemically unstable, is generated continuously to support delayed CaMKII activation during this period. Because prolonged Ang II action in cardiomyocytes requires NADPH oxidase-2^4^, but other cells can instead utilize NADPH oxidase-4 to mediate Ang II effects^21^, it will be of interest to identify the source of O_2_^.−^ for synthesis of ONOO^−^. Likewise, the source of NO remains to be determined. Of interest, all three NOS isoforms are present in cardiomyocytes, but NOS1 is uniquely upregulated by Ang II with a delay comparable to the second phase of CaMKII activation studied here^2^,^22^. Regardless of the sources of O_2_^.−^ and NO, the current results support the concept that Ca^2+^-independent and previously known Ca^2+^-dependent effects of ONOO^−^ act in concert to promote CaMKII activation *in vivo.*

These data reveal a novel signaling function for ONOO^−^. ONOO^−^ is typically viewed as a pathogenic mediator because ONOO^−^ generation (a) indicates the diversion of NO signaling away from guanylate cyclase activation and (b) yields potent oxidizing and nitrating species. Thus, the reaction of ONOO^−^ with proteins often inactivates or impairs function^9^. Therefore, the demonstration that ONOO^−^ can act as an intermediate in G-protein coupled receptor (GPCR) signaling by activating a functionally-significant downstream kinase defines a regulated and central biological action for this reactive species. Indeed, our results suggest that other receptor-NADPH oxidase effects that have been attributed to O_2_^.−^ or its dismutation product H_2_O_2_ may in fact be mediated by ONOO^−^.

This insight has implications for organ physiology, pharmacology and pathology. Previously, Ang II-NADPH oxidase signaling was primarily attributed to the actions of O_2_^.−^ and its dismutation product H_2_O_2_. Because Ang II and CaMKII oxidation are associated with cardiac pathology^23^,^24^ and ONOO^−^ is required for Ang II-induced CaMKII activation, suppressing ONOO^−^ generation and steady state concentrations in the heart may be of clinical benefit. It will also be interesting to determine whether this novel mechanism contributes to the clinical efficacy of Ang II AT1 receptor blockers (ARBs) and ACE (angiotensin converting enzyme) inhibitors. Furthermore, because Ang II-NADPH oxidase-Kv channel signaling occurs in neurons^7^,^25^ and ONOO^−^ is required for Ang II-Kv channel signaling in neuronal cells (Fig. 4), ONOO^−^ oxidation of CaMKII may be of significance for many organ and tissue functions.

## Methods

### All experiments were performed in accordance with relevant guidelines and regulations

#### Culture, transfection and treatment of cardiomyocytes and cell lines

Neonatal Sprague-Dawley rat ventricular cardiomyocyte cultures were generated by a protocol approved by the University of Pittsburgh Institutional Animal Care and Use Committee. Culture, transfection and Ang II treatments in serum-free medium (which yields nonbeating cardiomyocytes) were performed as previously described^2^,^5^. CATH.a cells were plated on 24 well plate with density of 1.25 × 10^5^ in RPMI-1640 medium (Invitrogen) plus 8% horse serum and 4% FBS. The following day cells were transfected using Lipofectamine Plus reagent (Invitrogen) with 0.5 μg DNA per well following the manufacturer’s protocol. After 4 hours, the medium was replaced with the fresh medium supplemented with 1 mM dibutryl-cAMP. Two days later, cells were treated with vehicle or Ang II for 6 hours in serum free medium. Neuro-2A cells were plated on 24 well plate at a density of 2 × 10^5^ per well in MEM (Invitrogen) with 10% FBS, 1 mM sodium pyruvate and 25 mM HEPES, pH 7.5. The following day, the cells were transfected using FuGENE HD Transfection Reagent (Promega) at a 4:1 ratio to DNA (0.75 μg per well) following the manufacturer’s protocol overnight. Then the medium was replaced with MEM 1% FBS for two days. Cells were then treated with vehicle or Ang II for 6 hours in serum free medium. H9C2 cells were plated on 24 well plate with density of 3.75 × 10^4^ per well in DMEM (Invitrogen) with 10% FBS. Cells were cultured 4 days and then were transfected using same method as used with Neuro-2A cells. The following day the medium was replaced with serum free medium and treated with vehicle or with Ang II for 24 hours.

Inhibitors were purchased from Sigma-Aldrich. 100 μM L-NAME (NG-Nitro-L-arginine methyl ester), D-NAME and 300 μM uric acid (UA) were applied for 30 minutes prior to treatment with 100 nM Ang II.

#### Immunoprecipitation (IP), SDS-PAGE and immunoblotting of endogenous *CaMKII.*

 IP and SDS-PAGE followed published protocols^4^. Membranes were incubated overnight at 4 °C with mouse monoclonal anti-T286 phospho-CaMKII antibody (1:500) (Santa Cruz Biotechnology) and rabbit polyclonal anti-total CaMKII (pan) (1:1000) (Cell Signaling) or with rabbit polyclonal anti-oxidized M281/282 CaMKII antibody (1:2000) (Millipore) and mouse monoclonal anti-total CaMKII antibody (1:1000) (Abcam). Membranes were washed 4 times 5 min with PBST and then incubated 1 hour in room temperature with IRDye 800CW Gt Anti-Rabbit IgG (H + L) antibody and IRDye 680LT Anti-Mouse IgG (H ± L) antibody (1:15,000) (Li-COR Bioscience). Infrared fluorescence was detected by the Odyssey Imaging System.

#### Nitrotyrosine detection

H9C2 cells treated with vehicle or Ang II (100 nM) in the presence or absence of L-NAME or UA as described above. Cells were harvested in lysis buffer (CelLytic M, Sigma) and proteins resolved by SDS-PAGE. Nitrotyrosine was detected by immunoblotting after overnight incubation with a 1:1000 dilution of a monoclonal antibody (clone 1A6, Millipore), followed by incubation with an Horse radish peroxidase-conjugated secondary antibody and chemiluminescent detection using the Immun-Star substrate system (BioRad). Densitometric analysis was performed using ImageJ software (NIH) and the resulting data was normalized to β-actin levels. Statistical evaluation was performed by one-way ANOVA with Tukey’s multiple comparisons test.

#### Peroxynitrite-induced oxidation of recombinant CaMKIIδ *in vitro*

Peroxynitrite was synthesized from nitrite and hydrogen peroxide using a quenched-flow reactor as described previously^14^. Excess hydrogen peroxide was removed using manganese dioxide and the concentration of the peroxynitrite solution was determined from its absorbance at 302 nm (ε = 1670 M ^−1^ cm^−1^) in 500 mM NaOH.

Recombinant human CaMKIIδ (EMD Millipore or purified as described previously^26^,^27^ was used for ONOO^−^-induced CaMKII oxidation. CaMKII oxidation in the presence of Ca^2+^/CaM was carried out in a reaction mixture containing 100 mM Tris, pH 7.0, 0.3–1 μM CaMKIIδ, with 6–10 μM of CaM and 0.5 mM of Ca^2+^, or in the absence of Ca^2+^/CaM and presence of 5 mM EGTA. ONOO^−^ was added last to the side of the reaction tube and rapidly mixed with the kinase by vortexing. For control reactions using the decayed ONOO^−^ product, the reaction was preincubated in the Tris pH 7.0 buffer 2–5 minutes to induce decomposition before addition of kinase. The ability of 100 mM Tris pH 7.0 to maintain the reaction pH was experimentally verified.

For activity measurements, 1 μM hCaMKIIδ wild type and mutants were oxidized as described above in 200 uM ONOO^−^ or 3 mM H_2_O_2_. Then 10–100 nM of the kinase was added to 50 mM PIPES, pH 7.0, 100 mM NaCl, 10 mM MgCl_2_, 100 μM ATP and 120 μCi/ml [γ-^32^P]ATP, 200 μM AC-2 peptide (KKALRRQETVDAL), and either 1 mM CaCl_2_ and 10 μM CaM for maximal activation as described previously^26^,^27^ or 5 mM EGTA (-Ca^2+^/CaM) for measuring activity independent of Ca^2+^/CaM. The reaction proceeded at 30 °C for 3 minutes (within the linear range) before spotting onto P81 filter paper and thoroughly washed in 75 mM phosphoric acid. The Ca^2+^/CaM independent activity was compared between the mutants and across the oxidative agents, as specific autonomous activity (Ca^2+^/CaM-independent activity divided by Ca^2+^/CaM-dependent activity). For activation assays comparing mutant proteins, CaMKII wild type and mutants were expressed in Hi5 cells using baculovirus. Hi5 cells expressing wildtype, double mutant (MM281, 282VV) and the triple mutant (MM281,282VV; C290V) were collected by centrifugation. The cells were lysed using a microfluidizer in buffer containing 50 mM HEPES, 100mM NaCl, 0.1 mM EDTA, and protease inhibitor with 500μM AEBSF HCl, 300 nM Aprotinin, 6 μM E-64, 20 μM Leupeptin. The lysate was then spun at 30000 rpm for 30 min and the supernatant was used to load onto a NiNTA resin. After binding, the resin was washed and the protein was eluted using buffer containing 50mM HEPES, 100 mM NaCl, 0.1 mM EDTA, 25 mM Imidazole. The elute was then passed through HiTrap desalting column and exchanged to buffer containing 50 mM Tris, 100 mM NaCl, 0.1 mM EDTA. The protein was quantified and 1 μM concentration of the protein was used for experiments as described above.

For M281/282 oxidation blots, 5–10 minutes after treatment with ONOO^−^ or dONOO^−^ at room temperature, samples were boiled in SDS-PAGE loading buffer and separated on a 8.5% acrylamide or 10% NuPage gel, which was transferred onto a Trans-Blot Transfer Medium Pure Nitrocellulose Membrane (0.2 μm) (Bio-Rad). Following blocking with bovine serum albumin or milk, the membrane was incubated overnight at 4 °C with anti-rabbit polyclonal against M281/282 oxidized-CaMKII antibody (1:2000) (Millipore) and anti-goat polyclonal antibody against total CaMKIIδ (Santa Cruz Biotechnology). Membranes were washed for 5 minutes 4 times with PBST or TBSt and then incubated 1 hour in room temperature with IRDye 800CW Gt Anti-goat IgG (H+L) antibody and IRDye 680LT Anti-rabbit IgG (H ± L) antibody (1:15,000) (Li-COR Bioscience). Infrared fluorescence was detected by Odyssey Imaging System.

#### Statistics

Data are quantified as the mean ± standard error of the mean (s.e.m.). Statistical significance was determined by Student’s t-test for experiments with two experimental conditions and one-way ANOVA followed by Dunnett’s, Bonferroni or Tukey’s post-test for experiments with more than two experimental conditions.

## Additional Information

**How to cite this article**: Zhou, C. *et al.* Novel Roles for Peroxynitrite in Angiotensin II and CaMKII Signaling. *Sci. Rep.* **6**, 23416; doi: 10.1038/srep23416 (2016).
